# Effects of the Long-Term Continuous Cropping of Yongfeng Yam on the Bacterial Community and Function in the Rhizospheric Soil

**DOI:** 10.3390/microorganisms11020274

**Published:** 2023-01-20

**Authors:** Jian Yao, Caiyun Wu, Linjuan Fan, Meihua Kang, Zirong Liu, Yuhui Huang, Xueliang Xu, Yingjuan Yao

**Affiliations:** 1 Institute of Agricultural Applied Microbiology, Jiangxi Academy of Agricultural Sciences, Nanchang 330200, China; 2 Ji’an Institute of Agricultural Sciences, Ji’an, 343103, China

**Keywords:** Yongfeng yam, continuous cropping, rhizospheric soil, bacterial community, network

## Abstract

Replant disease caused by continuous cropping commonly occurs in yam with consecutive monoculture. However, little is known about how the continuous cropping of yam affects the rhizospheric soil bacterial community structure. In this study, the effects of continuous cropping on rhizospheric soil characteristics, bacterial diversity, and community structure were investigated in the Yongfeng yam fields under monoculture for 1, 5, 10, 15, and 20 years. Long-term monoculture caused soil acidification and increased the concentration of available potassium (AK) and available phosphorus (AP), and soil bacterial richness, but decreased the soil bacterial diversity. An exception was for the field under monoculture for 20 years as it showed the highest bacterial diversity. The relative abundance of beneficial bacteria, such as Proteobacteria, Actinobacteria, and Chloroflexi decreased while the relative abundance of harmful bacteria, including Gemmatimonadetes and Acidobacteria, increased with an extended continuous cultivation time. The networks varied among yams with different cultivation years and became complex with the increase in cultivation years. However, after time in monoculture, the bacterial network decreased gradually and existed stably. These changes in bacterial community composition and co-occurrence of networks may increase the potential risk of soil-borne disease and reduce the yield and quality of Yongfeng yam.

## 1. Introduction

Yam (*Dioscorea* spp.) is an annual or perennial plant, originated in tropical and sub-tropical areas of the world, cultivated in Africa, South America, the Pacific, and Asia [1,2,3,4]. In China, Chinese yam (*D. polystachya*) has a long history of production, cultivation, and utilization, and it is cultivated from south to north areas [5]. Chinese yam has many varieties of cultivars such as Tiegun Yam, Ruichang Yam, Yongfeng Yam, and Taihe Yam [6]. The tubers of Chinese yam are rich in resistant starches, polysaccharides, steroidal sapogenins, minerals, and other nutritional components, so they are used not only as food but also as traditional Chinese medicine by locals for the treatment of diabetes, diarrhea, asthma, and other ailments [2,7,8,9]. Besides, the tubers of Chinese yam provide additional health benefits such as lowering blood sugar, regulating blood pressure, enhancing immunity, anti-aging, and anti-tumor mutation [9].

Due to the limited amount of arable land and a need to intensify regional agroindustrialization, Chinese yam has been continuously mono-cropped on a large scale. However, the monocropping of Chinese yam has caused serious soil-borne diseases, resulting in reduced yield or even no harvest, causing significant economic losses, which have seriously restricted the development of the Chinese yam industry. Based on previous reports, there are various factors contributing to the soil-borne diseases, including inappropriate farming operations, soil physicochemical property disorders, and the functional and structural imbalance of the soil microbial community [10,11]. Soil microorganisms are active in and vital for soil ecosystems and participate in transforming nutrients, such as decomposing organic matter, promoting nutrient cycling, and maintaining soil fertility [11]. Rhizosphere is the area near plant roots, and the bacterial community in this region is important for plant growth and health [12]. Recently, some studies have noted that long-term continuous cropping disrupted the rhizospheric bacterial community composition [10,13,14,15,16,17]. The shift of microbial composition not only affected the metabolic characteristics of the rhizosphere microbial community, but also resulted in a reduced representation of traits related to plant performance, such as nutrient metabolism and phytohormone biosynthesis [18].

However, little is known about the physicochemical properties of the soil and the microbial community in the rhizospheric soil of continuous cropping yams. At present, the research on yam diseases has mainly focused on the prevention and control of pests and diseases of the yam [2,19]. Therefore, in this study, high-throughput sequencing technology was used to study changes in the bacterial community in the rhizospheric soil of Yongfeng yams after different continuous cultivation years, and the correlation between soil physiochemical properties and microbial community diversity and composition after different continuous cultivation years was analyzed. The purpose of this study was to provide a theoretical basis for alleviating the obstacles of continuous cropping and to understand the mechanism of continuous cropping obstacles, which are important to guide agricultural practice.

## 2. Materials and Methods

### 2.1. Description of the Study Area and Experimental Design

The experimental site was located in Gannan experimental station of Yongfeng yam at Ji’an, Jiangxi province, China (N 27°19′16.59″, E 115°26′11.89″). This region has an average temperature of 18.5 °C, a mean annual precipitation of 1504 nm, and soil moisture of 10–12%. Up to 2021, fields that continuously planted with Yongfeng yam for 1, 5, 10, 15, and 20 years were selected and marked as YF_1Y, YF_5Y, YF_10Y, YF_15Y, and YF_20Y, respectively. Tubers of Yongfeng yam are planted on 10 March and harvested on 15 October each year. The field experiment plot followed a randomized block design with three replicates. The sowing density of Yongfeng yams was approximately 117,000 plants/a. The area of each experimental plot was 27 m^2^ with 316 plants. The amount of fertilizer applied was 1800 kg/ha of NPK compound fertilizer (N 15%, P_2_O_5_ 15% and K_2_O 15%) and 6000 kg/ ha of organic fertilizer (pH, 7–8; OM, 59.56 g/kg; TN, 12.48 g/kg; TP, 3.30 g/kg; TK, 9.05 g/kg).

### 2.2. Sample Collection and Soil Chemical Properties Detection

On October 15, 2021, five Yongfeng yam plants were harvested by the removal of the whole plant. The plants were shaken vigorously to remove soil in the root zone. Then the soil within 1–2 mm of the root was collected with brushing and combined to form one composite soil sample. Three soil samples were obtained from each treatment. A total of 15 soil samples were collected in self-sealing bags and temporarily stored on dry ice. After being transported to the laboratory, all samples were homogenized and sieved (2-mm mesh) to remove plant residues and stones. After that, each soil sample was divided into two parts: one was stored at −80 °C for bacterial community structure analysis, and the other was stored at 4 °C for the analysis of soil physicochemical properties.

### 2.3. Analysis of Soil Chemical Properties

The pH of rhizospheric soil sample was measured in 1:2.5 (*m/v*) mixture of soil and KCl solution with a pH meter (DZS-708L, Leici, shanghai, China) [20]. Briefly, 10 g air-dried soil sample mixed with 25 mL 1 mol/L KCl was shaken at 25 °C for 10 min (200 rpm), then kept stationary for about 1 h before measurement of the pH. Soil EC (soil/water suspension ratio: 1:5) was measured using an EC meter (DZS-708L, Leici, shanghai, China). The molybdenum-blue colorimetric method was used for available phosphorus (AP) analysis [21], and flame photometry (WGH6410, CHANGXI, China) was used for available potassium (AK) determination [22]. Soil NH_4_-N, NO_3_-N, and activities of β-glucosidase (GC), peroxidase (POD), leucine aminopeptidase (LAP), acid phosphatase (ACP), and N-acetyl-β-D-glucosidase (NAG) were determined using commercially available kits (Leier-bio, Hefei, China) following the manufacturer’s instructions. Soil chemical properties were determined in triplicate.

### 2.4. DNA Extraction, PCR Amplification, and High-Throughput Sequencing

Microbial DNA from different soil samples was extracted using the E.Z.N.A. ^®^Stool DNA Kit (Omega Biotek, Norcross, GA, USA) following the manufacturer’s instructions. The purity and concentration of DNA were detected by NanoDrop one Spectrophotometer (Thermo Scientific, Waltham, MA, USA). Amplification of V3–V4 region of the bacterial 16S rRNA gene with primers 338F (5′-ACTCCTACGGGAGGCAGCA-3′) and 806R (5′-GGACTACHVGGGTATCTAAT-3′) [20,23]. All amplifications were performed in 50 μL PCR mixtures that consisted of 25 μL Phusion^®^ High-Fidelity PCR Master Mix (NEB, Waltham, MA, USA), 1.5 μL of each primer (5 μM), and 100 ng of template DNA. The PCR procedure was set at 98 °C for 2 min; 30 cycles of 98 °C for 10 s, 53 °C for 30 s, 68 °C for 30 s; followed by a final extension at 72 °C for 10 min. The PCR products were checked by 2% agarose electrophoresis. After being purified and quantified, amplicons were standardized to equimolar levels, and pair-end sequencing was performed using the Illlumina NovaSeq platform at Bioyigene Biotechnology Co., Ltd. (Wuhan, China).

### 2.5. Sequence Analysis and Statistical Analysis

One -way analysis was used for multiple comparisons for all test parameters, and the variance was followed by Tukey’s honest significant difference multi-range test. Microbiome bioinformatics were performed with QIIME2 2021.11 according to the official tutorials (https://docs.qiime2.org/2021.11/tutorials/) [24] accessed on 3 December 2021. Sequence processing including quality filtering, denoising, merging, and the removal of chimeras which were performed using the DADA2 [25]. Non-singleton amplicon sequence variants (ASVs) were aligned with mafft [26] and used for constructing phylogeny with fasttree2 [27]. Chao1, Shannon, Simpson, Faith’s PD, and Good’s coverage indices were calculated using the ASV table in QIIME2. Beta diversity analysis was performed using weighted_unifrac distances [28] and visualized by principal coordinate analysis (PCoA). Veen diagram was generated to visualize the shared and the unique ASVs [29]. Differentially abundant taxa across groups were analyzed with Linear discriminant analysis effected size (LEfSe) [30]. Functional prediction analysis was carried out using FAPROTAX software [31].

Raw sequence data were deposited in NCBI database under accession number of PRJNA859533.

## 3. Results

### 3.1. Chemical Properties and Enzyme Activities of Rhizospheric Soil

The long-term continuous monoculture of yam changed the chemical properties of the rhizospheric soil, including concentrations of AP, AK, NH_4_-N, NO_3_-N, EC, and pH value. All soil samples were acidic in pH ranging from 5.1 to 4.1, and the pH value decreased with an increase in cultivation years. However, the concentration of AP and AK showed an increasing trend with the extension of cultivation years. Compared to YF_1Y treatment, AP and AK under YF_20Y treatment increased by 4.35 and 2.85 times, respectively. The EC value and the concentration of NH_4_-N fluctuated with the extension of continuous cropping time. The highest EC value (109.00 ± 1.63 μS/cm) and NH_4_-N (17.34 μg/g) were found in the YF_5Y treatment and YF_15Y treatment, respectively. The concentration of NO_3_-N was increased first and then decreased, reaching the highest value (39.71 μg/g) in the YF_15Y treatment (Table 1).

The enzyme activities of rhizospheric soil, including GC, POD, LAP, ACP, and NAG were tested. The activity of POD and LAP showed no significant change, while the activity of NAG increased along with the increase in continuous cropping years. Compared to the YF_1Y treatment, the activity of NAG under the YF_20Y treatment increased by 3.75 times. The activity of GC and ACP showed fluctuations along with the extension of cultivation years (Table 1), and the highest activities of GC (2.00 U/g) and ACP (6.36 U/g) were found in the YF_5Y treatment and YF_20Y treatment, respectively.

### 3.2. General Analysis of Sequencing Data

After filtering and chimera removal, 678,145 sequences in total were obtained from 15 samples and were clustered into 25,952 microbial ASVs using the DADA2 plugin. The average Good’s coverage was 97.91 ± 0.54% for the total bacterial community. The information of quality-filtered sequence is provided in Supplemental Table S1.

### 3.3. Alpha Diversity and Beta Diversity Analysis

**Figure 1:**
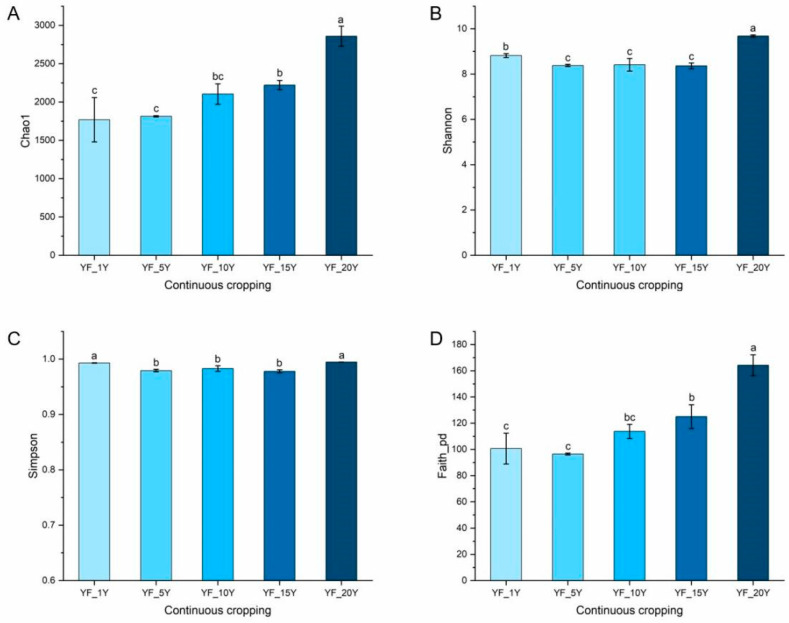
Changes in the alpha diversity indices of the bacterial community among different continuous cropping times of yam. (**A**) Chao1, (**B**) Shannon, (**C**) Simpson, (**D**) Faith_PD. Averages ± SD of samples in each group (with three biological replicates) were expressed in each column. Different letters within a row indicate significant differences at *p* < 0.05.

Continuous cultivation years significantly affected the bacterial richness (Chao1 index, *p* = 0.0003), phylogenetic diversity (Faith_PD index, *p* < 0.0001), and evenness (Shannon index, *p* < 0.0001, Simpson index, *p* = 0.0002) of the rhizospheric bacterial communities in the yam fields (Figure 1). Chao1 and Faith_PD indices for bacterial community greatly increased with the increase in continuous cultivation years. The Chao1 index increased from 1768.25 ± 289.97 to 2958.19 ± 130.69, while the Faith_PD index significantly increased from 100.65 ± 11.75 to 164.20 ± 7.93. However, the Shannon index decreased from 8.82 ± 0.081 to 8.36 ± 0.13 after 15 years of continuous cropping and then increased to 9.68 ± 0.045 after 20 years. The Simpson index decreased from 0.99 ± 0.00046 to 0.97 ± 0.0027 after 15 years of continuous cropping and then increased to 0.99 ± 0.00094 after 20 years.

**Figure 2:**
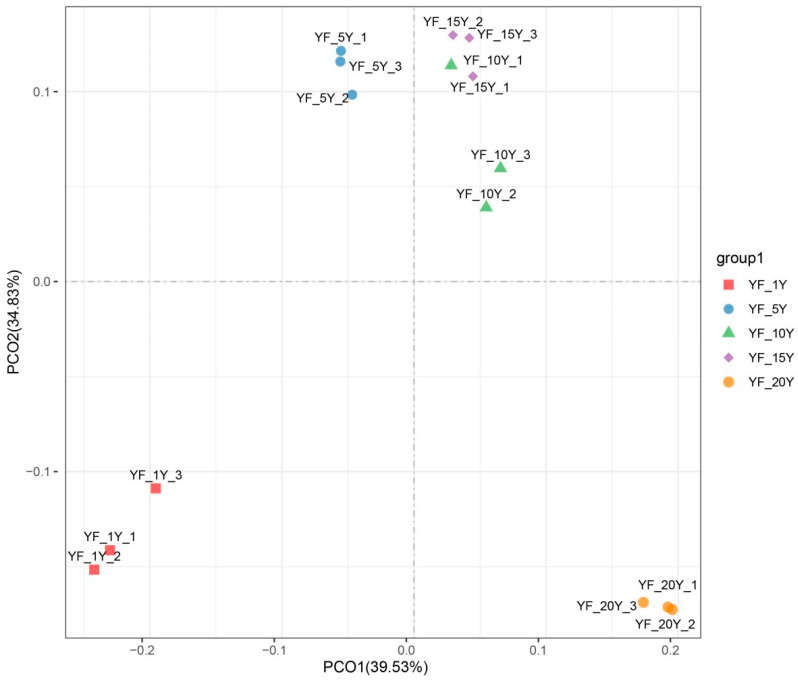
PCoA of bacterial communities in different rhizospheric soil samples based on weighted_unifrac distances.

To observe the similarities and differences in the bacterial community among different durations of continuous cultivation years, PCoA with weighted_unifrac distances were performed and visualized. PCoA depicted the degree of difference in bacterial community composition over different years of continuous cultivation. The first two principal coordinates explained 74.36% of the total variance, meaning that it well represented the characteristics of bacterial community composition. The results from all soil samples were well separated from each other. However, groups YF_5Y, YF_10Y, and YF_15Y were clustered together, indicating that the bacterial community compositions of YF_5Y, YF_10Y, and YF_15Y were similar, but differed greatly from the other two treatments, and the difference in the bacterial community composition between YF_1Y and YF_20Y was the greatest (Figure 2).

### 3.4. Taxonomic Composition Analysis

**Figure 3:**
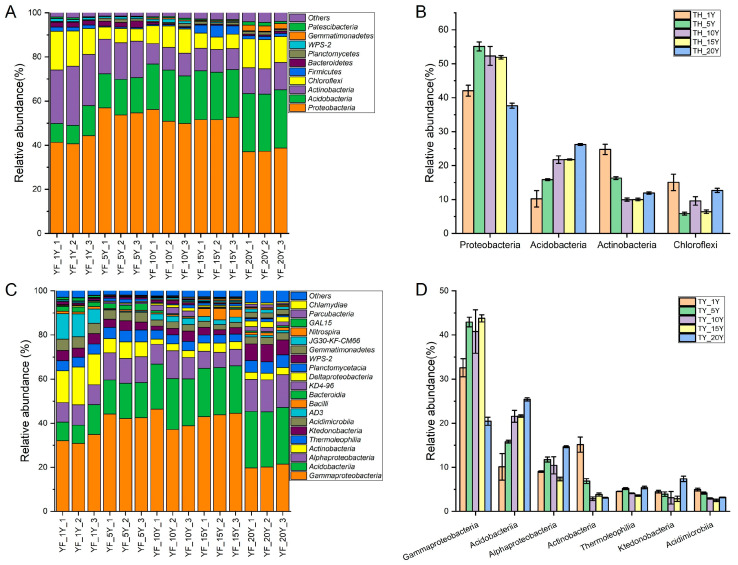
Relative abundance of bacterial communities. (**A**) Phyla are presented in bar plots showing the bacterial variation during different continuous cropping times of yam. (**B**) The relative abundance of the main phyla in soil samples. (**C**) Classes are presented in bar plots showing the bacterial variation during different continuous cropping times of yam. (**D**) The relative abundance of the main classes in soil samples.

A total of 30 phyla were identified through the taxonomic analysis of 16S rRNA sequences. Four phyla were dominant (relative abundance > 1%): Proteobacteria (from 42.1 to 55.1%), Acidobacteria (from 10.2 to 21.8%), Actinobacteria (from 10.0 to 24.8%), and Chloroflexi (from 5.9 to 15.1%) accounting for 90.1–93.6% of all sequences (Figure 3A). However, their relative abundances were different under different continuous cultivation years. For instance, the abundance of Acidobacteria increased with the extension of continuous cultivation years, while Actinobacteria showed the opposite trend. The abundance of Proteobacteria increased up to 15 years of continuous cropping, then decreased after 15 years (Figure 3B).

At the class level, 16 classes had an abundance >1%. The dominant taxonomic groups included Gammaproteobacteria (from 20.4 to 43.8%), Acidobacteriia (from 10.1 to 25.4%), Alphaproteobacteria (from 7.4 to 14.4%), Actinobacteria (from 2.9 to 15.2%), Thermoleophilia (from 3.6 to 5.4%), Ktedonobacteria (from 2.9 to 7.4%), and Acidimicrobiia (from 2.5 to 5.0%). The relative abundances of Oxyphotobacteria, Melainabacteria, and Blastocatellia (Subgroup_4) were less than 0.1%. Most groups had relative abundances <1% (Figure 3C). The relative abundance of Gammaproteobacteria increased first and then decreased with the increasing continuous cultivation time. The relative abundance of Acidobacteriia increased while the abundance of Actinobacteria decreased with the extension of continuous cultivation years. The abundances of Thermoleophilia, Ktedonobacteria, and Acidimicrobiia decreased or changed little over time (Figure 3D).

### 3.5. Differences in Microbial Community among Different Continuous Cultivation Years

**Figure 4:**
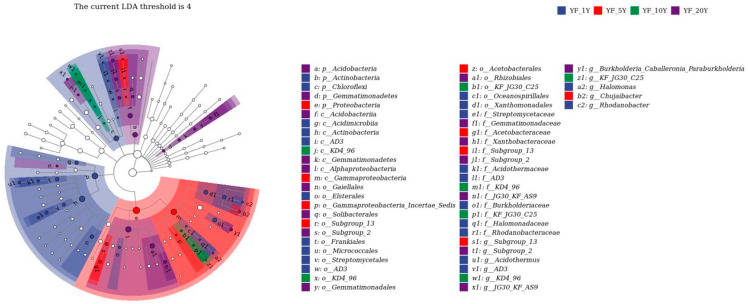
The LEfSE analysis of differences in bacterial abundances among different treatments with a threshold value of 4.0. The taxonomic clades map showed the taxonomic rank relationships of the major taxa in the sample community from phylum to genus (from inner circle to outer circle). Node size corresponds to the average relative abundance of the taxon. Hollow nodes represent taxon with no significant differences between groups, while nodes with other colors (blue for YF_1Y, red for YF_5Y, green for YF_10Y, and purple for YF_20Y) indicate that these taxa showed significant differences between groups, and their abundance was higher in the grouped samples represented by this color. Letters identify taxon names that differed significantly between groups.

To find differences in bacterial communities of all soil samples, Veen diagrams were generated based on ASVs: 11,865 bacterial ASVs were detected in all samples, among which 2238, 1705, 1840, 2122, and 3816 ASVs were detected specifically in samples from YF_1Y, YF_5Y, YF_10Y, YF_15Y, and YF_20Y, respectively, and 144 ASVs were shared by all samples (Supplemental Figure S1). LEfSE was performed to identify discriminatory biomarkers (LDA scores of >4) (Figure 4). The results showed two phyla including Actinobacteria and Chloroflexi; three bacterial classes including Acidimicrobiia, Actinobacteria, and AD3; six orders including Frankiales, Micrococcales, Streptomycetales, AD3, Xanthomonadales, and Oceanospirillales; and six families including Streptomycetaceae, Acidothermaceae, AD3, Burkholderiaceae, Halomonadaceae, and Rhodanobacteraceae had higher relative abundances in the YF_1Y sample. Phylum Proteobacteria, class Gammaproteobacteria, and three families including Acidobacteriaceae_Subgroup_1, Acetobacteraceae, and Subgroup 13 had higher relative abundances in the YF_5Y sample. The discriminant biomarkers enriched in the YF_10Y sample included members from the class KD4_96 and family KF_JG30_C25, while the discriminant biomarkers enriched in YF_20Y sample included members from two phyla Acidobacteria and Gemmatimonadetes; three classes from Acidobacteriia, Alphaproteobacteria, and Gemmatimonadetes; and five orders from Subgroup 2, Rhizobiales, Gemmatimonadetes, Gaiellales, and Solibacterales.

### 3.6. Relationship between Environmental Factors and Bacterial Community Structure

**Figure 5:**
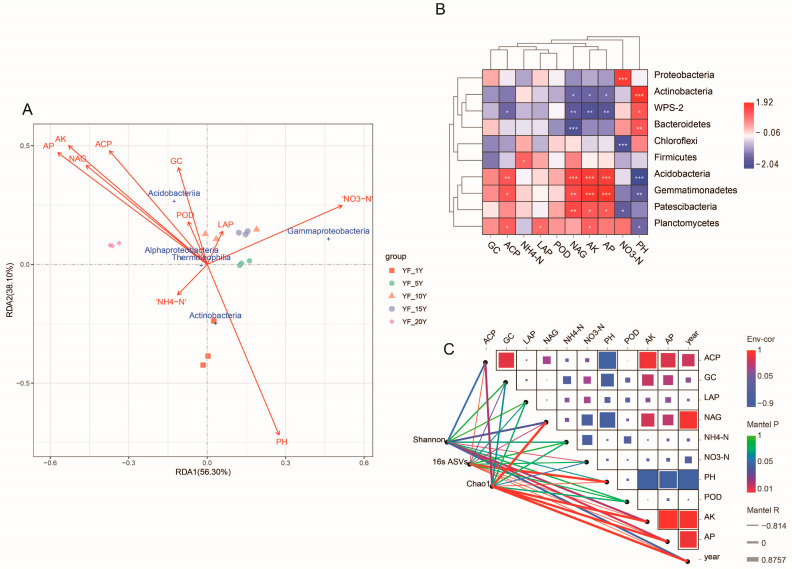
Analysis of the relationship between environmental factors and microbial community structure. (**A**) The redundancy analysis (RDA) of bacterial communities and soil properties in different treatments. (**B**) Correlations between the 10 dominant bacterial phyla and soil properties. * *p* < 0.05; ** *p* < 0.01; *** *p* < 0.001. (**C**) The relationship between environmental factors and soil bacterial community composition. Pairwise comparison of environmental factors and color gradients representing Pearson’s correlation coefficient. The 16s ASVs, Chao I, and Shannon were related to each environmental factor using the Mantel test. Edge color revealed the statistical significance based on significance. Edge width corresponded to the Mantel’s statistic for the corresponding distance correlations. AK: available potassium; AP: available phosphorous; GC: β -glucosidase; ACP: acid phosphatase; POD: peroxidase; LAP: leucine aminopeptidase; NAG: N-acetyl -β -D-glucosidase.

In order to clarify the main environmental factors affecting bacterial community composition, RDA analysis was performed (Figure 5A). The first two RDA axes explained 56.30% and 38.10% of the total variation, respectively. According to the importance of their effects on bacterial community composition, the tested soil properties were ranked as follows: soil pH > AP >AK > NAG > ACP > NO_3_-N > GC > POD > NH_4_-N > LAP. The results of the Spearman’s correlation coefficient analysis revealed that AP (*p* < 0.001), AK (*p* < 0.01), ACP (*p* < 0.01), and NAG (*p* < 0.001) were significantly positively correlated with the relative abundance of Acidobacteria, while AP (*p* < 0.01), AK (*p* < 0.01), NAG (*p* < 0.01), and ACP (*p* < 0.05) were significantly negatively correlated with WPS-2. The dominant phylum Proteobacteria was obviously positively correlated with NO_3_-N (*p* < 0.001), while Chloroflexi was significantly negatively correlated with NO_3_-N (*p* < 0.001). pH was significantly positively correlated with Actinobacteria (*p* < 0.001), Bacteroidetes (*p* < 0.01), and WPS-2 (*p* < 0.05), while it was obviously negatively correlated with Acidobacteria (*p* < 0.001) and Gemmatimonadetes (*p* < 0.01). Bacteroidetes (*p* < 0.001), WPS-2 (*p* < 0.01) and Actinobacteria (*p* < 0.05) were obviously negatively correlated with NAG (Figure 5B).

In order to identify the main ecological divers that influenced the composition of bacterial communities, the distance-corrected dissimilarities of 16s ASVs, Shannon, and Chao I with environmental factors were analyzed (Figure 5C). Mantel test analysis revealed that the bacterial community was significantly impacted by the continuous cultivation years (Chao I r = 0.88, *p* < 0.001; 16s ASVs r = −0.80, *p* < 0.001). Soil nutrients such as AP (Shannon r = 0.60, *p* < 0.05; 16s ASVs r = −0.79, *p* < 0.001; Chao I r = 0.80, *p* < 0.001) and AK (Shannon r = 0.58, *p* < 0.05; 16s ASVs r = −0.82, *p* < 0. 001; Chao I r = 0.80, *p* < 0.001) impacted the bacterial communities. Furthermore, NAG (Shannon r = 0.53, *p* < 0.05; 16s ASVs r = 0.59, *p* < 0.05; Chao I r = 0.82, *p* < 0.001) was also found to impact the bacterial communities.

### 3.7. Network Analysis of Soil Bacterial Community

**Figure 6:**
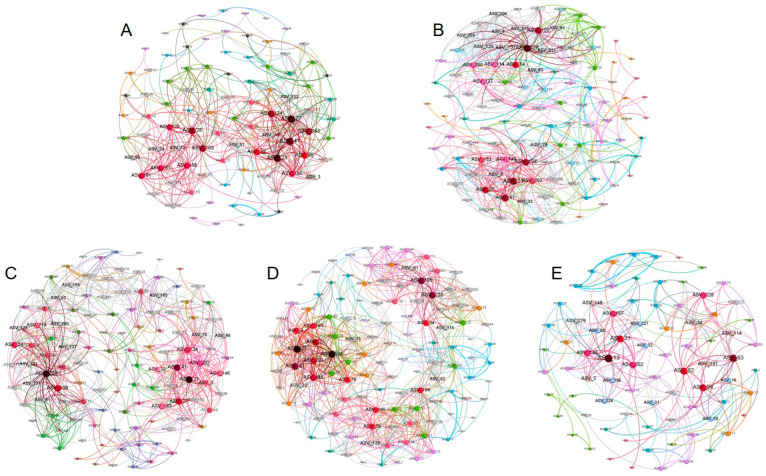
Co-occurrence network of the soil bacterial community for YF_1Y (**A**), YF_5Y (**B**), YF_10Y (**C**), YF_15Y (**D**), and YF_20Y (**E**) treatment. Nodes represent ASVs, edge indicats a significant positive correlation between the two ASVs, the size of the node represents the number of edges connected to the node, the thickness of the edge represents the degree of correlation, and the same color represents the same module.

In order to decipher the potential interactions among microbial taxa, a co-occurrence network based on 16s ASVs level showed the relationship between bacteria with different cultivation years (Figure 6). The topological features of the molecular ecological networks showed that YF_1Y, YF_5Y, YF_10Y, and YF_15Y had more nodes and edges than YF_20Y. Compared with one year of cultivation, the number of nodes increased by 33.7% after 15 years of continuous cultivation, then decreased by 13.0% after 20 years of continuous cultivation. The number of edges showed a similar trend; compared with one year of cultivation, it increased by 83.0% after 15 years of continuous cultivation, then decreased by 36.2% after 20 years of continuous cultivation. The value of clustering coefficient and network density of the ecological network increased and became stable, then decreased after 20 years of continuous cultivation. The percentage of negative correlations in YF_1Y (22.2%), YF_5Y (25.0%), YF10Y (25.5%), YF15Y (30.8%), and YF_20Y (32.9%) was less than the positive correlations, and the percentage of negative correlations increased with the extension of continuous cultivation. For the keystone species, ASV21 (*Edaphobacter*_sp) and ASV27 (unclassified_*Chujaibacter*), ASV228 (uncultured_*planctomycete*) and ASV21 (*Edaphobacter*_sp), ASV228 (uncultured_*planctomycete*) and ASV21 (*Edaphobacter*_sp), ASV21 (*Edaphobacter*_sp) and ASV228 (uncultured_*planctomycete*), and ASV13 (uncultured_forest) were identified in YF_1Y, YF_5Y, YF_10Y, YF_15Y and YF_20Y networks, respectively.

### 3.8. Prediction of Bacterial Community Function

**Figure 7:**
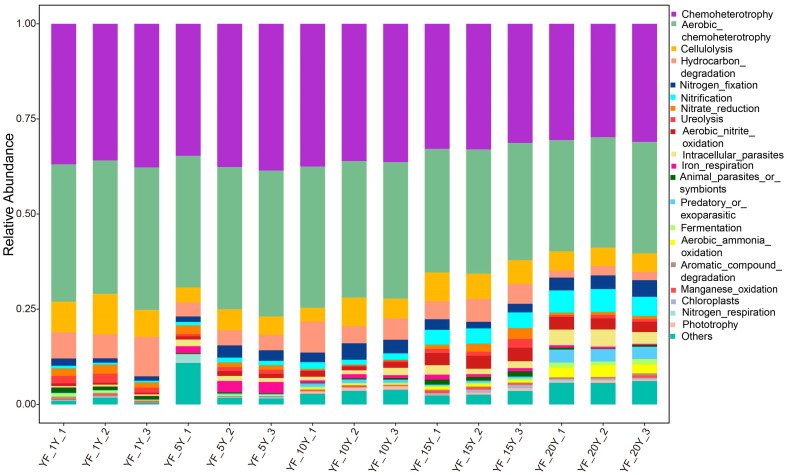
Stacked bar chart of dominant function group.

To study the functional difference of bacterial communities in samples with different continuous cropping years, FAPROTAX software was used to perform a functional prediction (Figure 7). Moreover, the results showed that the main functions of the bacterial community were chemoheterotrophy, aerobic_chemoheterotrophy, cellulolysis, nitrification, hydrocarbon_degradation, nitrogen_fixation, predatory_or_exoparasitic, and aerobic_nitrite_oxidation in all samples. However, chemoheterotrophy (*p* < 0.001), cellulolysis (*p* < 0.001), hydrocarbon_degradation (*p* < 0.001), and aerobic_chemoheterotrophy (*p* < 0.001) functions significantly declined with the extension of continuous cropping years, and nitrification (*p* < 0.001) and aerobic_nitrite_oxidation (*p* < 0.05) significantly increased with the extension of continuous cropping years.

## 4. Discussion

Recently, knowledge on the effects of continuous cropping on the rhizospheric microbes of different plants has increased [32,33,34]. However, no study has yet investigated the dominant microbial community in rhizospheric soil during long-term yam monocultures. To comprehensively understand the rhizospheric soil bacterial community structure of Yongfeng yam in different continuous cultivation years, the Illumina NovaSeq method was used. These results revealed that the bacterial richness increased with the extension of continuous cultivation years, which is consistent with previous finding with peanut [35], *Andrographis paniculate* [36], and *Rehmannia glutinosa* [37], but is contrary to findings regarding sweet potato [38], *Panax notoginseng* [39], and *Lycium barbarum* [40]. The results of bacterial diversity (Shannon index) decreased first, remained stable, then increased with long-term continuous cropping, indicating a decrease in bacterial diversity up to 15 years of continuous cropping and an increase in bacterial diversity after 20 years. This might be due to changes in the proportion of dominant bacteria in different planting years, resulting in changes in the evenness of bacterial structure. Changes in richness and evenness ultimately led to the changes in bacterial diversity. This result does not agree with previous studies on sweet potato [10,38,41], rice [42], cotton [43], *Lycium barbarum* [40], and peanut [35], where bacterial diversity increased or decreased monotonically with continuous cropping time. However, a higher bacterial richness and bacterial diversity were found over 30 years and 10 years cropping with the highest for 30 years cropping of maize [44]. Furthermore, PCoA analysis revealed that the continuous cropping of the Yongfeng yam significantly affected bacterial communities, which was consistent with Pang et al. who found that the continuous cropping of sugarcane for different times also obviously affected the bacterial community [14]. This phenomenon was also observed in the continuous cropping of sweet potato [38], peanut [35] and alfalfa [45]. Overall, these results indicated that continuous cropping had certain effects on the composition of the rhizospheric soil bacterial community. Differences in bacterial richness and diversity found in different studies may be caused by variations in the duration of continuous cropping, planting pattern, soil environmental conditions, plant types, and other factors.

Results of the phylogenetic analysis showed that Proteobacteria, Acidobacteria, Actinobacteria, and Chloroflexi were the dominant phyla in all samples. The phylum Proteobacteria plays a key role in phylogenetic, ecological, and disease-inhibiting functions, and it is the most dynamic taxon associated with rhizoctonia disease suppression. In addition, it is involved in organic matter decomposition and plant growth promotion [10,35]. In our study, the most dominant bacterial phylum in all samples was Proteobacteria, which generally agreed with previous studies [10,38,46]. However, as the continuous cultivation time increased over 5 years, the relative abundance of Proteobacteria decreased, which was almost consistent with Gao et al. [10], but inconsistent with studies on sweet potato [38] and black pepper [47]. Acidobacteria was the most abundant bacterial phylum in soils with nutrient limitation [48]. Long-term continuous cropping leads to soil fertility reduction, which increases the relative abundance of Acidobacteria [35]. A similar phenomenon appeared in this study, where the relative abundance of Acidobacteria increased significantly (*p* < 0.0001) with the extension of continuous cultivation years. Actinobacteria was reported to be one of the major taxa to prevent some soil-borne diseases, mainly by producing antibacterial, antifungal, and nematocidal compounds and agents. Actinobacteria are also strongly enriched in suppressive soils. However, its relative abundance decreases considerably with the extension of continuous cultivation years [38]. A 30-year-old tea plantation had an obviously lower soil pH, which in turn decreased the abundance of Actinobacteria and Chloroflexi [49]. In this study, both Actinobacteria and Chloroflexi all decreased with the extension of continuous cultivation time. Conversely, Gemmatimonadetes, a harmful bacterium that leads to N loss and reduced crop growth, increased with the continuous cropping of Yongfeng yam, which was consistent with the effect observed in sweet potato [10]. In addition, the abundance of *Nitrospira* was increased with the extension of continuous cultivation years in our investigation. It was reported that *Nitrospira* was positively correlated with Fusarium wilt, speculating that *Nitrospira* might produce some substances that are beneficial to the infection of *Fusarium* [50]. All these results indicated that the change of the abundance of these bacteria might affect the change of plant disease resistance. The imbalance between the proportion of beneficial bacteria and harmful bacteria may lead to the transformation of healthy soil into pathogenic soil.

Soil enzyme activity is the most important index reflecting soil quality and fertility. Moreover, the activity of soil enzymes is related to the nutrient and microbial community of cultivation years. Peroxidase can decompose hydrogen peroxide, reducing its effect on organisms. No significant difference in soil peroxidase activity after continuous cropping of Yongfeng yams was found, which was the same as the research of Liu et al. [51] on Hami melon (Cucumis melo var. saccharinus). Phosphatase can promote the transformation of organic phosphorus to inorganic phosphorus in soil and plays an important role in the accumulation of soil available phosphorus. Except for YF_5Y, the activity of acid phosphatase increased obviously after continuous cropping in this study. It was also found that the continuous cropping of konjac increased acid phosphatase activity [52]. In addition, the chemical properties of rhizospheric soil were also changed after long-term continuous cropping. In our study, the concentration of AP and AK showed an increased trend with the extension of continuous cultivation time, which may have been because of the overuse of inorganic fertilizer or the insufficient utilization of nutrients in the soil by Yongfeng yam after a long period of monoculture. Shao et al. found that the AP and AK contents increased significantly with increasing continuous cropping of peanuts [53]. It was reported that the overuse of inorganic fertilizer significantly decreased soil pH [54], which was confirmed in our study. Soil pH is a key factor affecting soil bacterial diversity and community composition; soil pH affects soil microbial physiological metabolism, alters the competitive relationship within microbial communities, and inhibits the growth of unadapted microbes [10,38]. Of the four bacterial phyla with the highest relative abundance, Acidobacteria was negatively correlated with soil pH while Actinobacteria was positively correlated with soil pH, which was the opposite of the findings of Gao et al. [10]. Furthermore, Gemmatimonadetes was also negatively correlated with soil pH. Gemmatimonadetes and Acidobacteria were positively correlated with AK and AP. Mantel test analysis revealed pH, AK, and AP were the main factors affecting the composition of the soil bacterial community and this might be the reason that the composition of bacterial communities of YF_5Y, YF_10Y, and YF_15Y were more similar as they showed a more similar soil pH and concentration of AP and AK. Our investigation demonstrated that the soil parameters were impacted significantly by continuous cropping, showing that continuous cropping modified their soil characteristics, such as soil acidification and the accumulation of AK and AP, and subsequently changed their bacterial communities. Moreover, a healthy soil microbial community can be sustained by improving soil properties through increasing the use of bioorganic fertilizers and reducing the use of fertilizers.

The co-occurrence patterns and network analysis provided a more detailed understanding of the interactions among soil bacterial taxa, which play an important role in ecosystem process and function. In a constructed network, the positive interaction mostly results from commensalism, while negative interaction is due to competition, predation, amensalism, and so on [55]. In this study, the network negative correlations of YF_20Y were higher than in other treatments, suggesting that long-term continuous planting of Yam strengthened the competitive relationship between bacteria. Meanwhile, due to the highest modularity of YF_20Y, its network stability was stronger in all treatments, indicating that a more competitive relationship between bacterial interactions will enhance the stability of the community [56,57,58]. However, the number of nodes and edges, density, and average clustering coefficient of YF_20Y were the lowest in all treatments, indicating that YF_20Y showed the lowest network complexity. In addition, compared with YF_1Y and YF_20Y, YF_5Y, YF_10Y, and YF_15Y revealed a similar number of nodes and edges, average degree, average clustering coefficient, and density, indicating that YF_5Y, YF_10Y, and YF_15Y showed a similar network complexity. Moreover, the complexity of the network presented a “sample-complex-sample” form. Furthermore, by classifying the keystone taxa in the network, we found that Acidobacteria (ASV21 and ASV13) existed in all networks. We hold the opinion that Acidobacteria plays an important role in all networks, and it was found that YF_1Y, YF_5Y, YF_10Y, and YF_15Y had the same keystone ASV of Acidobacteria member (Subgroup_1), and this ASV was inconsistent with YF_20Y (Subgroup_2), indicating that long-term cultivation of Yongfeng yams changed the Acidobacteria members with important functions in the network. All these results revealed that maybe 15 years was the longest continuous cultivation time of Yongfeng yams in the same field. It may be time to grow another crop to improve the soil properties. However, this continuous cultivation period may be changed with fertilization regimes, pest control, and other factors, which needs further verification in the future.

## 5. Conclusions

In conclusion, the continuous cropping of the Yongfeng yam changed the physiochemical features of rhizospheric soil and the bacterial community structure, diversity, and functional structure, including reduction in soil pH, decline main functions of chemoheterotrophy, and aerobic_chemoheterotrophy, decreased abundance of potentially beneficial bacteria, and increased abundance of harmful bacteria. Furthermore, the networks differed among different continuous cultivation years of yams. All these changes might reassemble the soil microbial community and could lead to increased Yongfeng yam disease levels in continuous cropping systems. Soil pH, AK, and AP content were important factors that influenced the structure of the rhizospheric soil bacterial community and should be targeted to solve the problem of continuous cropping obstacles and the growth of the Yongfeng yam.

## Figures and Tables

**Table 1:** Soil physiochemical properties.

	YF_1Y	YF_5Y	YF_10Y	YF_15Y	YF_20Y
pH	5.1 ± 0.04 a	4.5 ± 0.09 b	4.4 ± 0.04 bc	4.3 ± 0.04 c	4.1 ± 0.04 d
EC (μS/cm)	64.03 ± 2.15 d	109.00 ± 1.63 a	71.50 ± 0.60 c	94.46 ± 0.99 b	54.501.25 e
AP (mg/g)	1.74 ± 1.01 c	4.68 ± 0.78 bc	3.73 ± 0.65 bc	5.15 ± 1.25 b	9.31 ± 0.78 a
AK (mg/kg)	82.16 ± 8.57 d	189.59 ± 10.07 b	153.09 ± 10.14 c	198.49 ± 14.93 b	316.32 ± 7.85 a
NH_4_-N(μg/g)	15.75 ± 4.96 a	12.77 ± 0.88 a	11.54 ± 0.61 a	17.34 ± 1.38 a	15.45 ± 0.46 a
NO_3_-N(μg/g)	13.86 ± 4.48 b	39.71 ± 0.82 a	23.61 ± 1.73 b	23.81 ± 7.39 b	12.65 ± 1.48 b
GC (U/g)	0.18 ± 0.12 c	2.00 ± 0.31 a	1.29 ± 0.65 ab	0.57 ± 0.05 bc	1.76 ± 0.09 a
POD (U/g)	1.58 ± 0.93 a	1.40 ± 0.82 a	2.84 ± 2.20 a	1.91 ± 0.46 a	2.00 ± 0.26 a
LAP (U/g)	1.31 ± 0.69 a	2.40 ± 1.25 a	0.95 ± 0.40 a	2.59 ± 1.00 a	1.90 ± 0.89 a
ACP (U/g)	5.09 ± 0.09 d	6.10 ± 0.29 ab	5.60 ± 0.09 c	5.71 ± 0.06 bc	6.36 ± 0.02 a
NAG (U/g)	0.40 ± 0.19 c	0.17 ± 0.10 c	0.93 ± 0.11 bc	1.55 ± 0.34 ab	1.90 ± 0.52 a

AP: available phosphorus; AK: available potassium; NH_4_-N: ammonium nitrogen; NO_3_-N: nitrate nitrogen; GC: β-glucosidase; POD: peroxidase; LAP: leucine aminopeptidase; ACP: acid phosphatase; NAG: N-acetyl-β-D-glucosidase. Values are the mean± standard deviation of triplicate determinations. Different letters in the same row indicate a significant difference among all treatments tested by one-way ANOVA (*p* < 0.05).

## Data Availability

All data generated or analyzed during this study are included in this published article.

## Supplementary Materials

The following supporting information can be downloaded at: https://www.mdpi.com/article/10.3390/microorganisms11020274/s1, Table S1: The number of 16SrDNA sequences and sampling good’s coverage of soil microbiome; Figure S1: Veen diagram showing the number of unique bacterial detected in different continuous cropping time.
