# Supplementary material for: Effects of the Long-Term Continuous Cropping of Yongfeng Yam on the Bacterial Community and Function in the Rhizospheric Soil
Source: Microorganisms. 2023 Jan 20;11(2):274. doi: 10.3390/microorganisms11020274 (PMC9959641; doi:10.3390/microorganisms11020274)

Supplement Table and Figure

Table S1 The number of 16SrDNA sequences and sampling good's coverage of soil microbiome

Samples	No. of raw sequences	No. of clean sequences	Good's coverage (%)	No. of ASVs
YF_1Y_1	80478	67,040	98.66	10,416
YF_1Y_2	75511	68,591	99.09	10,441
YF_1Y_3	77454	47,912	97.87	10,686
YF_5Y_1	63583	39,637	98.23	10,926
YF_5Y_2	67142	45,273	98.26	11,120
YF_5Y_3	70106	48,117	98.24	11,282
YF_10Y_1	65966	46,378	98.08	11,441
YF_10Y_2	71281	47,778	97.90	11,640
YF_10Y_3	70649	45,970	97.71	11,848
YF_15Y_1	63030	38,816	97.66	12,121
YF_15Y_2	62733	40,150	97.83	12,342
YF_15Y_3	65959	42,286	97.64	12,568
YF_20Y_1	64713	34,843	97.05	13,020
YF_20Y_2	66329	35,430	97.00	13,479
YF_20Y_3	61063	29,924	97.45	13,887

Figure S1 Veen diagram showing the number of unique bacterial detected in different continuous cropping time.

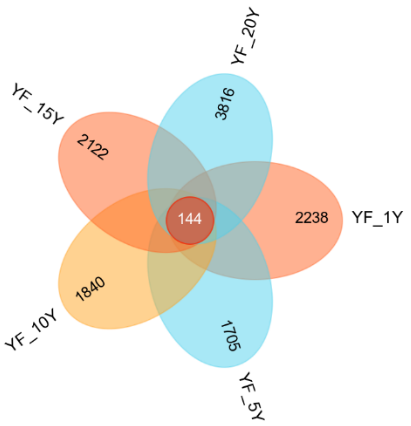

Supplement: Supplementary file 1 [file microorganisms-11-00274-s001.zip › microorganisms-2072054-supplementary.pdf]
